# Identification of CXCL10-Relevant Tumor Microenvironment Characterization and Clinical Outcome in Ovarian Cancer

**DOI:** 10.3389/fgene.2021.678747

**Published:** 2021-07-27

**Authors:** Jing Jin, Yi Li, Tobias Achu Muluh, Liangke Zhi, Qijie Zhao

**Affiliations:** ^1^Department of Oncology, The Second People’s Hospital of Yibin, Yibin, China; ^2^Department of Oncology, The Affiliated Hospital of Southwest Medical University, Luzhou, China; ^3^Sichuan Jinxing Education Consulting Co., Ltd., Chengdu, China; ^4^Department of Pathophysiology, College of Basic Medical Science, Southwest Medical University, Luzhou, China; ^5^Department of Radiologic Technology, Faculty of Associated Medical Sciences, Chiang Mai University, Chiang Mai, Thailand

**Keywords:** CXCL10, tumor microenvironment, immune infiltration, survival, genetic alteration

## Abstract

**Background:**

Chemokines are implicated in tumor microenvironment (TME) cell infiltration. Development of ovarian cancer involves heterologous cells together with the adjacent microenvironment. Nonetheless, our understanding of the chemokine-related TME characteristics in ovarian cancer remains obscure.

**Methods:**

In this large-scale multi-platform study of 10 microarray datasets consisting of 1,673 ovarian cancer patients, we comprehensively evaluated CXCL10 and CXCL9 expression risk classifications for predicting overall survival (OS) and TME immune characteristics. The cross-validation between a standard cohort (TCGA: The Cancer Genome Atlas) and three test cohorts (GEO: Gene-Expression Omnibus) was applied. We investigated differences in the biological functions and the underlying mechanisms between high- and low-risk classifications.

**Results:**

We identified that evaluation of CXCL10 expression could predict the tumor development, immune cell infiltration, TME signature, genetic alteration, and patient prognosis in ovarian cancer. Low-risk classification was characterized by high CXCL10 expression and prolonged prognosis, which was positively associated with specific immune cell infiltration (i.e., T cells, DCs, aDC, and Th2 cells) and TME immune-relevant signatures. Meanwhile, the high-risk classification was defined by lower CXCL10/CXCL9 expression and relevant poor prognosis and immune infiltrations. The CXCL10-based low-risk classification was also linked to antitumor biological function of specific immune gene sets, such as IL2-STAT5 signaling. Additionally, a mutational pattern featured by enrichment of C > T transition was further identified to be associated with immune cell infiltration.

**Conclusions:**

This work proposed a promising biomarker for evaluating TME immune characteristics and clinical outcomes in patients with ovarian cancer. Estimation of CXCL10 risk pattern sheds a novel insight on ovarian cancer TME immune characteristics and provides strategies for ovarian cancer immunotherapy.

## Introduction

Ovarian cancer is one of the most lethal gynecological tumors with complex mechanisms, and more than 295,000 new cases and 184,000 deaths were estimated to be reported in 2018 (Longacre et al., 2016; Bray et al., 2018). Generally, the majority of females with ovarian cancer are diagnosed at an advanced stage, because there are no early-stage symptoms and reliable screening strategies for early monitoring (Levanon et al., 2008; Wei et al., 2013). Genomic investigation has been the primary methodology used in modern efforts to discover relevant biological targets of ovarian cancer (Wei et al., 2013; Hillman et al., 2018). It also provides a powerful scientific basis for solid molecular functions in different clinical parameters, such as overall survival (OS), tumor pathobiology, and grades (Fehniger et al., 2019). Notably, the tumor microenvironment (TME) plays an important role in tumor progress and therapy (Nwani et al., 2018; Zhang et al., 2018; Greppi et al., 2019). Moreover, accumulating evidences suggested the significance of TME-related molecular and dysregulated signaling pathways in both ovarian cancer cells and immune cells (Browning et al., 2018; Drakes and Stiff, 2018) and revealed that the interrelated gene signatures are more important than genomic factors at the single-cell level (Au et al., 2017; Curtis et al., 2018). High-throughput technologies paved a way for us to evaluate multiple targets and predict clinical patients’ TME (Singer and Anderson, 2019). Genomic alteration in genes is involved in expression change and associated with resident cell type composition within the disordered TME (Porpiglia et al., 2004; Fridman et al., 2017; Desrichard et al., 2018). The identification of multiple TME characteristics might be a hopeful strategy to predict the progress of patients with ovarian cancer.

The immune system has been deemed a decisive factor during ovarian cancer initiation and development (Knutson et al., 2015). Ovarian cancer is characterized by deregulation of immune surveillance (Greppi et al., 2019), and immunotherapy can strongly benefit from the intervention of the immune checkpoint (Topalian et al., 2015; Nagarsheth et al., 2017). Regulating the transport of immune cells by chemokines in the tumor is implicated in shaping immune regulatory cell presentation in TME (Knutson et al., 2015; Dangaj et al., 2019). Recently, several studies have analyzed the involvement of chemokine function in ovarian cancer TME. The CXCR3^+^ T effectors were observed to have been abolished in ovarian cancers, resulting in the collateral limitation of efficient antitumor immunity (Redjimi et al., 2012). The CXCL9 or CXCL10/CXCR3 axis regulates immune cell migration, differentiation, and activation, leading to tumor suppression, and a better understanding of potential mechanisms is necessary to develop effective cancer control (Tokunaga et al., 2018). CXCL10 expression is correlated with antigen processing and tumor-infiltrating lymphocyte (TIL) infiltration in ovarian cancer and positively associated with patient’s OS (Bronger et al., 2016; Au et al., 2017). However, CXCL10 can also mediate worse prognosis in some entities and chemotaxis of tumor-promoting cells, such as regulatory T cells (Tregs) (Redjimi et al., 2012). The chemokine-related TME immunological signature remains a potential to be applied in ovarian cancer. To date, the comprehensive landscape of CXCL10-related immune cell infiltration and TME characteristics in ovarian cancer have not been elucidated.

In this study, based on visual inspection curves of estimated survival dependent on CXCL10 and CXCL9 expression, we integrated multiple datasets with gene expression, which contained 1,673 cases in total to explore ovarian cancer TME immunological characteristics from immune signature gene sets. Two proposed computational algorithms were applied to estimate the fraction of 23 immune cell types and quantify TME infiltration patterns (TME score) through four independent datasets. CXCL10 was found to be a robust prognostic biomarker and verified to have a high predictive power for ovarian cancer TME immunological characteristics.

## Results

### Landscape of Ovarian Cancer Survival and Clinicopathological Characteristics of CXCL10 and CXCL9 Expression

**FIGURE 1:**
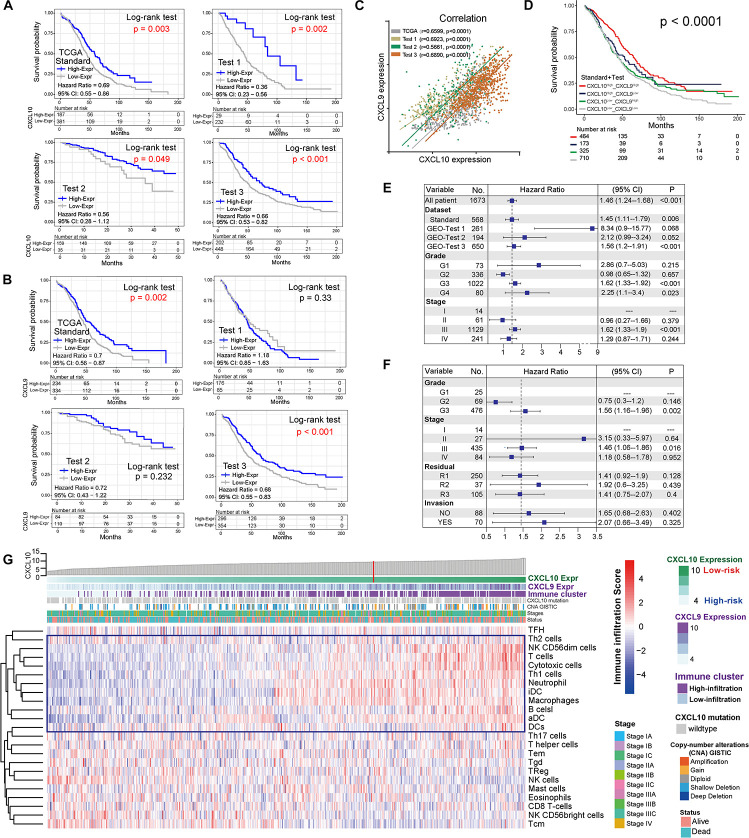
Landscape of the CXCL10 and CXCL9 expression and TME cells-infiltration in ovarian cancer. **(A)** Kaplan-Meier survival analysis of CXCL10 expression in four independent cohorts of ovarian cancer. Patients were stratified into low- (blue; CXCL10 high expression) and high- risk (gray; CXCL10 low expression) classifications with a cutoff value of OS by survminer. Log-rank *P* < 0.05 showed a significant survival difference. **(B)** Kaplan-Meier survival analysis of CXCL9 expression in four independent cohorts of ovarian cancer. **(C)** Correlation analysis between CXCL10 and CXCL9 expression in four independent cohort. The *r* values were verified by the Pearson test (*P* < 0.0001). **(D)** Survival analyses for subgroup patients stratified by both CXCL10 and CXCL9 expression with Kaplan-Meier curves (*P* < 0.0001, Log-rank test). **(E)** Forest plot representation of the clinical prognostic value between high/low risk classification in independent cohorts and clinical parameters. Hazard ratios (HR) > 1.0 indicated that CXCL10 expression is a favorable prognostic biomarker. **(F)** Subgroup analyses estimated prognostic value of tumor pathology in standard cohort. **(G)** Heat map showed the scoring of TME immune cell infiltration in the standard cohort according to CXCL10 and CXCL9 expression subgroups. The thick line represented the unsupervised clustering of positive correlation. The risk classifications, immune cluster subgroups, Copy-number alteration (CNA), survival status and stages were used as patient annotations.

CXCL10 and CXCL9 differential expression profiles of 1,673 patients [mean months ± SD, 42.1 ± 34.2; 8% early stage (I, II), 92% late stage (III, IV)] in four independent cohorts were analyzed (Table 1). The patients in different cohorts were stratified according to CXCL10 and CXCL9 expression-related OS, and the cutoff value was determined by the survminer package. Overall, in four independent cohorts, we found that a lower CXCL10 expression was significantly associated with poorer prognosis (high risk) [standard cohort HR range: 0.69 (95% CI: 0.55–0.86; *P* = 0.003)]. Thus, we stratified the standard cohort and three test cohort patients into high-risk (lower CXCL10 expression) and low-risk groups (higher CXCL10 expression), respectively, by visually inspecting the curves of estimated survival (Figure 1A). Meanwhile, the lower CXCL9 expression was significantly associated with poorer prognosis in the standard (*P* = 0.002) and Test 3 groups (*P* < 0.001) (Figure 1B). The CXCL10 and CXCL9 expression levels showed a positive correlation in four independent groups of ovarian cancer (Figure 1C). Not surprisingly, combination group survival analysis revealed that patients with a high expression of CXCL10 and CXCL9 showed better prognosis (Figure 1D). Next, we verified the consistency of the three independent cohorts and the standard cohort by the multivariable Cox model (HR = high vs low risk) after adjusting the datasets, stages, and grades. The prognostic index of CXCL10-based high-risk classification in three test cohorts was obviously higher than that of The Cancer Genome Atlas (TCGA) standard cohort (Figure 1E). Although the results of subgroup analysis were heterogeneous, high-risk classification was positively associated with malignant neoplastic grades (G2 to G4) and pathological stages (II to III) in the merged group (standard plus three test cohort patients). We also observed that tumor invasion, residual status, and grades in the standard cohort were positively associated with the high-risk classification (Figure 1F).

**TABLE 1:** Summary of the four cohorts included in the study.

Cohorts	Platform	Samples	Datasets
Standard	Affymetrix Human Genome U133A Array	568	TCGA
Test 1	Affymetrix Human Genome U133A Array	261	GSE3149, GSE14764, GSE23554, and GSE26712
Test 2	ABI Human Genome Survey Microarray Version 2	194	GSE49997
Test 3	Agilent-014850 Whole Human Genome Microarray 4 K × 44 K G4112F	650	GSE17260, GSE32062, GSE53963, and GSE73614

In order to further characterize and understand the tumor immunobiology with CXCL10 expression, the construction scheme of the ovarian TME cell infiltration pattern was systematically evaluated. We evaluated the association between CXCL10 expression and immune cell populations from transcriptomic data (Figure 1G). Unsupervised hierarchical clustering of 568 samples with matched TME cell microarray probes in the standard cohort was presented. The heatmap depicted a strong correlation between high CXCL10 expression and T cells, cytotoxic cells, T helper 1 (Th1) cells, and different DCs. Likewise, tumor with low CXCL10 expression displayed a general lack of immune infiltration. Moreover, the same observation made across all test cohorts was also examined (Supplementary Figure 1).

### CXCL10/CXCL9 Traits and Infiltrating Patterns of TME

**FIGURE 2:**
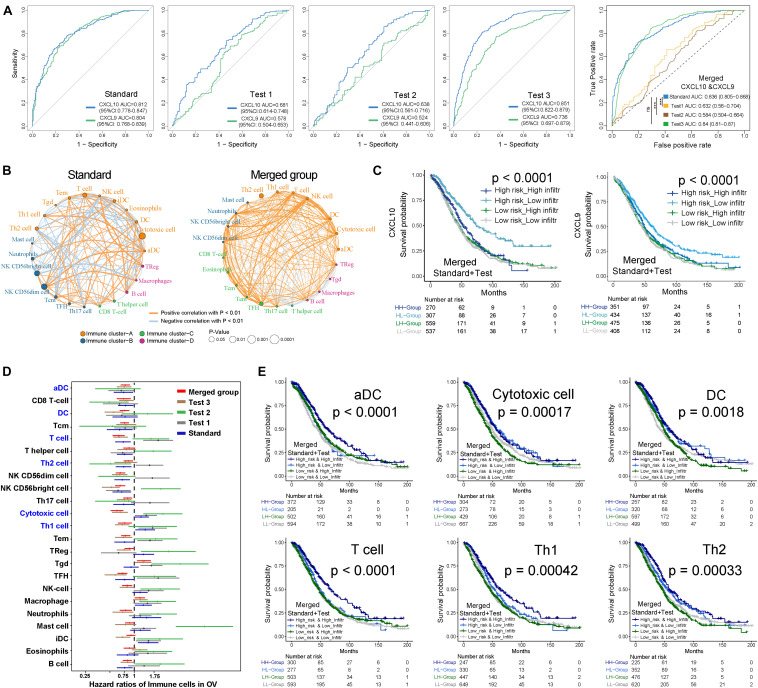
Kaplan-Meier survival analyses of TME cell infiltration and in distinct risk classification. **(A)** ROC curves measuring the predictive value of the CXCL10 and CXCL9 in high/low risk of immune infiltration of ovarian cancer. In four independent groups (Left), blue line represented the CXCL10 and green lines represented the CXCL9. Comparison of ROC curves for the ability of the combined-CXCL10/CXCL9 to estimate infiltration level of four group of ovarian cancer datasets (Right). **(B)** The interaction between TME immune cell types in standard cohort (left) and merged sample (right). The strength of correlation was presented with thickness of lines, and positive correlation is marked with orange and negative correlation with light-blue. Red dot represented favor for OS, and black dots represented risk for OS. Four immune clusters were presented, namely, clusters (A–D). **(C)** Kaplan-Meier curves for the merged patients with complete information in ovarian cancer were stratified by both CXCL10/CXCL9 risk classification (high/low) and TME infiltration cluster (high/low) (Log-rank test, *P* < 0.0001). **(D)** Prognosis analyses of difference TME immune cells in ovarian cancer. Estimated survival-cut point in each subset of five groups was plotted with 95% confidence intervals (CI) lines. **(E)** Kaplan-Meier curves based on the prognosis of infiltration (high/low) and CXCL10 risk classification (high/low), infiltration impact was defined in panel **(D)** in all tumor samples. Six types of immune cells (T cells, DCs, aDC, Th1, Th2, and Cytotoxic cells) were selected. *P* values were indicated in the graphs.

Through the pROC package analysis (Robin et al., 2011), we observed that CXCL10 showed a predictive advantage in the infiltration group when compared to CXCL9 in four independent groups of ovarian cancer (Figure 2A, left). In addition, combining CXCL10 and CXCL9 improved the predictive value compared with that of CXCL10 or CXCL9 alone in merged samples (Figure 2A, right). Of note, the CXCL10/CXCL9 showed a robust prediction in the standard group compared with the other two groups (likelihood ratio test, *P* < 0.0001). Thus, CXCL10 presented a prior role in ovarian cancer when compared to CXCL9. To determine the optimal cluster immune cell types, the TME cell network depicted a comprehensive landscape of high-risk and low-risk classifications, including TME immune cell interactions and cell lineages of patients with ovarian cancer (Figure 2B). Four TME cell infiltration subtypes were shown in schematics (immune clusters A, B, C, and D). Moreover, cluster A was characterized by correlating with the infiltration of CXCL10-related immune cells in both standard and merged groups. However, the patients showed inconsistent survival with two intrinsic phenotypes of CXCL10/CXCL9-related risk and immune cell infiltration (log-rank *P* < 0.001), which might be attributed to the incongruous function of immune cells in TME (Figure 2C). Given that the key role of individual immune cells in TME can mediate tumor development and predict clinical outcome, we presented 23 types of TME immune cell infiltration as another survival marker in different cohorts of ovarian cancer (Figure 2D). As CXCL10-related risk was correlated with TME immune cell infiltration, we also explored the prognostic value of the merged group. Notably, patients with high-infiltration TME immune cells [DCs, Th1, T helper 2 (Th2), and T cells] and low-risk groups showed a synergistic function in improving OS (log-rank *P* < 0.01) (Figure 2E).

**FIGURE 3:**
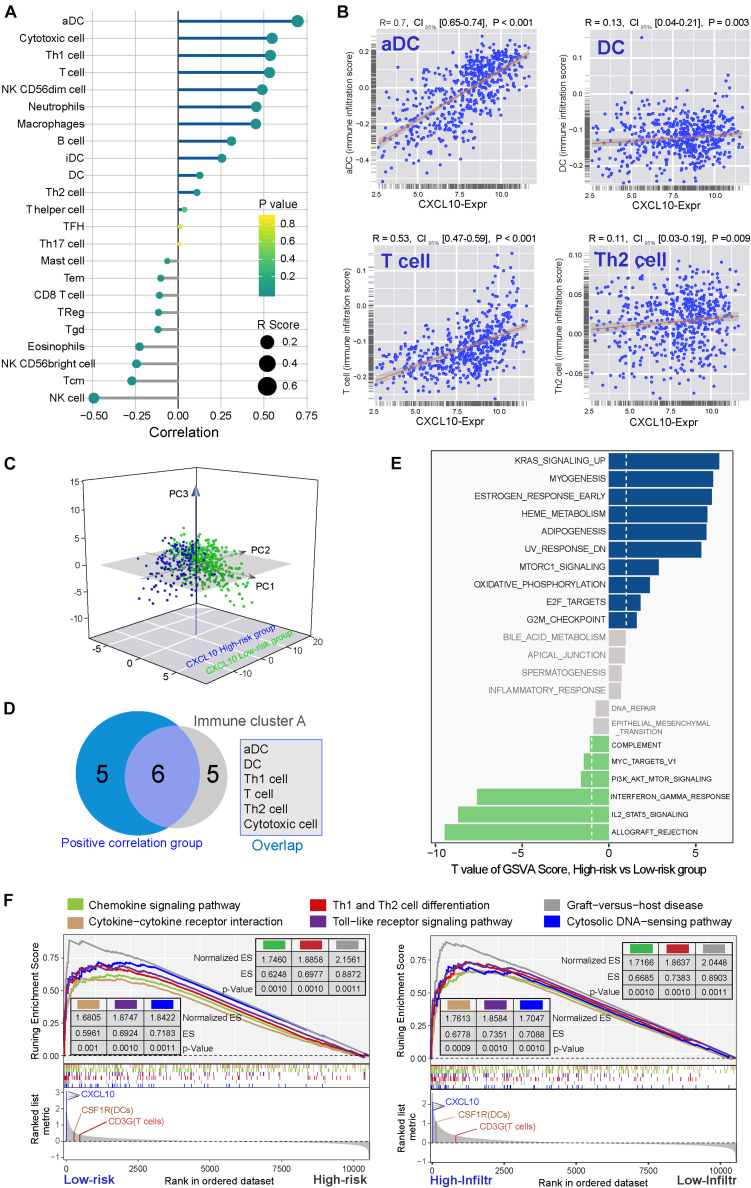
TME immune cells transcriptome traits in TCGA-Ovarian Cancer. **(A)** Correlation between CXCL10 and known TME immune cell infiltration in standard cohort was analyzed by Spearman. Positive correlation was marked with right arms (blue), and negative correlation was marked with left arms (gray). The circles size and color were represented the R score and *P* value. **(B)** Scatter plots depicted the positive correlation between CXCL10 expression and four particularly interested immune cells. **(C)** Venn diagram showed the overlap of immune cells between immune cluster-A and CXCL10 positive correlation group. We filtered six cells to further analysis CXCL10-related underlying mechanism. **(D)** PCA was used for the expression profiles of high- and low-risk classifications to distinguish selected immune signature gene sets. These signature genes expression was well distinguished based on the different risk classifications. High risk classification marked with blue, and low risk classification marked with green. **(E)** Differences in pathway activities. The 135 selected immune signature genes expression between high-risk and low-risk classifications were analyzed by GSVA. T values are from a linear model, ultimately correcting the effects from the patient of origin. The KRAS signaling-up was marked in high-risk classification, and IL2-STAT5 signaling was marked in low-risk classification. **(F)** GSEA of the whole genome expression data from TCGA ovarian cancer in low- to high-risk classification and high- to low-infiltration patients. The enrichment results with immune associations between low-risk classification and high TME cells-infiltration are shown. *P* values were determined by using the Kolmogorov–Smirnov test.

In addition to patient OS, TME immune cell infiltration tendency, intergenic relationship, and enriched biological processes were also associated with CXCL10 expression. In the standard cohort, as a whole, cytotoxic cells, INF γ-DC (aDC), DCs, Th1, Th2, and T-cell infiltration degrees were positively associated with CXCL10 expression (Figure 3A), and significantly positive correlations were revealed between CXCL10 expression and aDC, DCs, Th2, and T-cell infiltration values (Figure 3B). Furthermore, through overlapping of the immune cells in cluster A and the positive correlation group (Figure 3C), CXCL10 expression was mostly correlated with six of these cells’ infiltration, namely, aDC, DCs, Th1 cells, Th2 cells, T cells, and cytotoxic cells. When taking into account these selected immune cell probes, we utilized a PCA plot to show that samples were clustered primarily by transcription profile in different groups. A closer inspection revealed that the probe expression pattern possessed an obvious separation between high- and low-risk classifications in the standard cohort (Figure 3D). Despite individual variability, the graphics showed appreciable immune cell genetic dysregulation in different risk classifications of ovarian cancer.

Furthermore, we evaluated the enrichment of these selected immune probes in tumor biological function. Gene set variation analysis with two different groups (high risk and low risk) were analyzed by the GSVA package of R software (Hanzelmann et al., 2013), based on the KEGG and Gene Ontology (GO). A direct comparison of these immune probes in high-risk and low-risk classifications revealed that KRAS signaling upregulation (| log fold change| = 0.297, adjusted *P*-value < 0.0001) was the top enriched signature in high-risk ovarian cancer (Figure 3E). Meanwhile, low-risk-group immune cell signature genes were significantly associated with allograft rejection and IL2-STAT5 hallmark signaling (| log fold change| = 0.346, adjusted *P*-value < 0.0001). Furthermore, we used gene set enrichment analysis (GSEA) to perform all ovarian cancer transcription analyses from low risk to high risk and high infiltration to low infiltration (Figure 3F). We found that both the low-risk group with a higher CXCL10 expression and the high-infiltration group were closely connected to immune cell function [chemokine signal (ES > 0.62, *P*-value = 0.001), cytokine receptor interaction (ES > 0.5, *P*-value < 0.001), Th1/2 differentiation (ES > 0.69, *P*-value = 0.001), and cytosolic DNA-sensing pathway (ES > 0.7, *P*-value = 0.001)].

### Tumor Genomic Characteristics Associated With Immune Cell Infiltration

**FIGURE 4:**
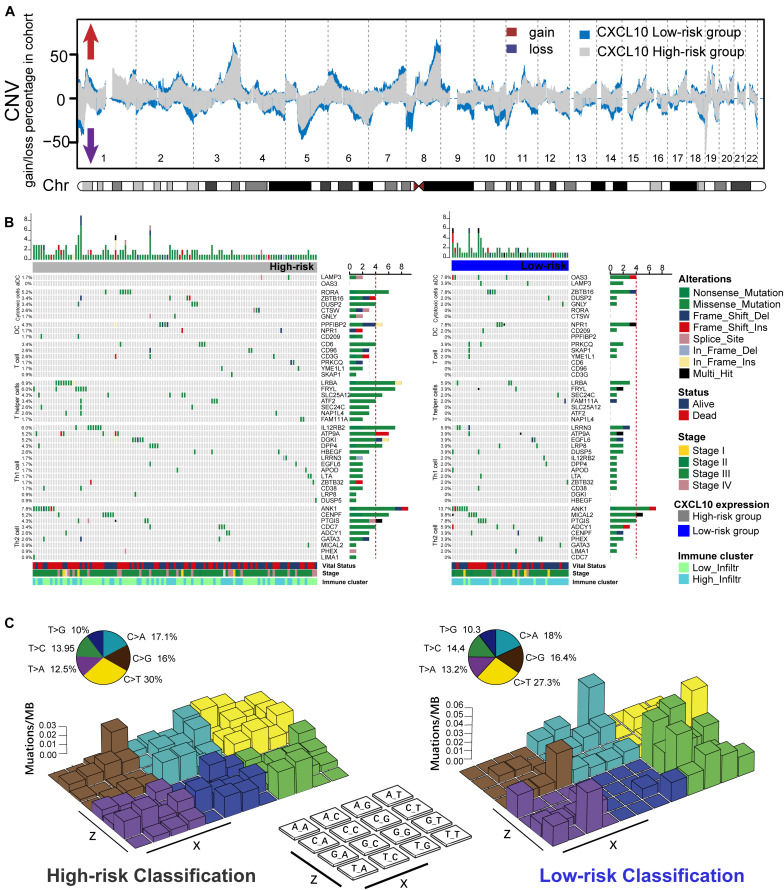
Genetic alteration extracted from the aggregated ovarian cancer samples. **(A)** The Copy number profiles of high (blue)- and low (gray)-risk classification showed different pattern of gains and losses. Frequency of copy number alterations were plotted on Y-axis, and corresponding chromosome was plotted on X-axis (chr 1–22). **(B)** Mutational landscape of specific immune-cell genetic alterations in high-risk classification (left) and low risk classification (right). The middle panel depicted 45 valid signature genes alteration across analyzed TCGA dataset, and different alteration types were coded with different color. The number on the right indicated the mutation frequency of each gene. The right panel indicated the proportion of each variant type. Each column represented individual patients, and displayed with survival status, stages, and immune cluster. **(C)** Legoplot representation of mutation patterns in two classifications. Single-nucleotide substitutions are divided into six categories with 16 surrounding flanking bases (each category represented by a different color). Inset pie showed the proportion of six categories of mutation patterns.

In terms of underlying mechanisms, we tried to investigate genetic instability on transcription. Based on genomic data in the standard cohort, we comprehensively analyzed the genetic alteration and single-nucleotide polymorphism (SNP) in ovarian cancer. We compared copy-number variations (CNVs) between high-risk and low-risk groups’ whole-genome sequencing data from TCGA tumor samples. In general, we observed that low-risk groups showed higher CNV in coverage (Figure 4A). Next, somatic alteration distributions of six immune cell signature genes from the high-risk group and low-risk group were analyzed (Figures 4B and Supplementary Figure 2B). A median of 162 alterations per sample (ranging from 20 to 1,922) in a total of 40,924 coding somatic alterations from 165 valid patient profiles was employed. We found that higher somatic tumor alteration load was obviously associated with high-risk classification patients. In addition, through the comparison between high- and low-risk classifications’ genetic alteration frequency, cytotoxic cell (RORA: 5.2 vs 0%; DUSP2: 3.4 vs 2%; CTSW: 2.6 vs 0%), DCs (PPFIBP2: 4.3 vs 0%), T cells (CD3G: 2.6 vs 0%; CD94: 2.6 vs 0%; CD6: 3.4 vs 0%), Th1 cells (IL12RB2: 6.0 vs 2.0%; ATP9A: 5.2 vs 3.9%; DGKI: 5.2 vs 0%; DPP4: 4.3 vs 2.0%; HBEGF: 2.6 vs 0%) and Th2 cell (CENPF: 5.2 vs 3.9%; CDC7: 3.4 vs 0%; CD3G: 2.6 vs 0%) infiltrations were most probably accompanied with altered genetic functions in high-risk classifications.

Moreover, the overall mutational pattern of these selected samples was dominated by C > T, and alteration patterns were similar between the high-risk and low-risk classifications Figure 4C). Meanwhile, the C > T and T > G average alteration frequency was higher in high-risk patients than in low-risk patients (Supplementary Figure 2C). We extracted five mutational signatures (i.e., signatures 1, 3, 5, 8, and 25) from the above data (Supplementary Figure 2A). The five signatures were annotated against the COSMIC signature version 2 (Alexandrov et al., 2013). Signature 1, featured by C > T transitions at CpG dinucleotides, is thought to be connected with age-related accumulation of spontaneous deamination of 5-methylcytosine. Notably, signature 1 was negatively associated with patient outcomes in human cancer, including ovarian cancer (Essers et al., 2020). The identification of this mutational signature might provide a new perspective to study the mechanism of chemokine-related TME formation and explanation of rising tumor incidence, as well as exploring individual mutations and their roles in cancer immunity and immunotherapy (Table 2).

**TABLE 2:** Summary of CXCL10-based subgroups.

Characteristics	High-risk	Low-risk
Expression	**−**	**+**
Overall survival	**−**	**+**
Immune infiltration	**+**	**−**
TME signature	**−**	**+**
Genetic alteration	**+**	**−**

### Construction of the TME Phenotypes and Clinical Functional Annotation

**FIGURE 5:**
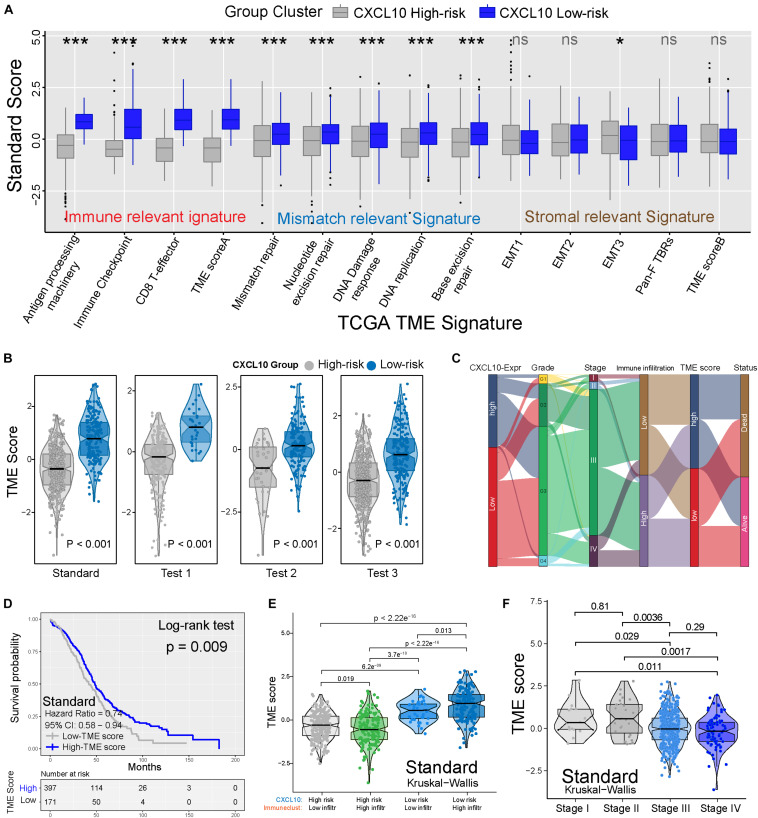
TME characteristics with risk classification and tumor development. **(A)** TME different signatures (immune-relevant signature, mismatch-relevant signature and stromal-relevant signature) score between high- and low-risk classification. The lines in the boxes represented median value. The asterisks represented the statistical *P* value (*P < 0.05; **P < 0.01; ***P < 0.001). **(B)** Differences of TME score in high- and low-risk classification from four independent cohorts. **(C)** Sankey diagram showed the link of high- and low-risk classifications, grades, stages, immune cell infiltration, TME scores, and survival outcomes. **(D)** Kaplan-Meier curves indicated that higher TME score was significantly associated with better OS in standard cohort (*P* = 0.009, Log-rank test). **(E)** Differences in TME scores were positively associated with CXCL10-related immune cell infiltration. Patients with different TME score were stratified by both CXCL10 risk classification (high/low) and TME infiltration cluster (high/low). The Kruskal-Wallis test was applied to compare the differences between every two groups (*p* < 0.0001). **(F)** Differences in TME scores among different pathological stages of ovarian cancer. The thick line represented the median value. The differences between every two groups were analyzed by Kruskal-Wallis test. (*p* < 0.05).

To better clarify the functionality of the TME signature of CXCL10-mediated risk in ovarian cancer, we tested known TME signatures (Mariathasan et al., 2018) in four independent cohorts (Supplementary Figure 3A). These analyses confirmed that low-risk ovarian cancer patients were positively associated with immune-relevant signatures (antigen-processing machinery, immune checkpoint, and CD8^+^ T effector) (Figure 5A and Supplementary Figure 3A). Consistent with these results, low-risk ovarian cancer was notably linked to higher TME scores (Figure 5B). The Sankey diagram showed the comprehensive correlation of the different clinical parameters, TME characteristics, and ovarian cancer risk (Figure 5C). When examining the association between TME score and survival in four different cohorts, we found that a higher TME score was significantly associated with prolonged OS (Figure 5D and Supplementary Figure 3B). Moreover, in the standard cohort, TME scores remained statistically significant after taking into account CXCL10-related risk classification and pathological stages. As expected, consistent with the outcomes of CXCL10-related infiltrating patterns, the patients with higher immune cell infiltration and CXCL10 expression were positively associated with TME score (Figure 5E and Supplementary Figure 4A). The predictive value of TME score to tumor development was also confirmed in the standard (*n* = 568) and merged cohorts (*n* = 1,486) with valid pathological stage data, and we found that a decreased TME score was associated with tumor deterioration (Figure 5F and Supplementary Figures 4B,C).

## Discussion

In this study, we carried out an analysis of 1,673 ovarian cancer patients from 10 valid studies and identified the expression patterns of CXCL10 and CXCL9 together with their related TME immune characteristics. We revealed that CXCL10 expression tended to be positively associated with prognosis and play a prior role in TME immune characteristics in certain stages and most samples of ovarian cancer. Based on functional analysis of specific immune signature genes, our observation suggested that different risk classifications were accompanied with distinct gene expression patterns and biological function. Mechanically, the CXCL10-based high-risk group was independently associated with elevated immune genetic alteration and C > T transversions.

Chemokine-related responses to favor or suppress antitumor immunity were involved in various TMEs (Viola et al., 2012), including ovarian cancer (Rainczuk et al., 2012; Knutson et al., 2015). The establishment of predictive biomarkers for TME is essential to maximize the immunotherapy benefit (Duan et al., 2018). Recently, therapeutic modulators that induce CXCL10 and CXCL9 expression were reported to improve immune cell responses and TME in ovarian cancer (Bronger et al., 2016; Au et al., 2017). Accumulating evidence indicated that CXCL10 plays a crucial role in ovarian cancer, such as regulating tumor progression, TIL infiltration, and associated gene expression (Yang et al., 2012; Bronger et al., 2016; Au et al., 2017; Santos et al., 2020). Despite existing data having greatly increased our understanding of CXCL10, sufficient validation of this biomarker is still limited. Therefore, we generated four independent cohorts that covered the most common microarray platforms, ultimately elucidating the comprehensive landscape of CXCL10 in ovarian cancer clinical outcomes, TME characteristics, and underlying mechanisms. With the help of several computational algorithms, we quantified the population-specific TME cell infiltration and TME signatures based on relevant gene sets, both of which were significantly associated with CXCL10-related risk in ovarian cancer.

Current studies have indicated that some types of chemokine accumulation were associated with good prognosis, including CXCL10 and CXCL9 (Peng et al., 2010; Muralidhar and Barbolina, 2013; Bronger et al., 2016). Moreover, CXCL10 has been reported to be an antitumorigenic chemokine (Tanese et al., 2012). This finding is consistent with prior studies that demonstrate better outcomes related to CXCL10 expression and support the hypothesis of inhibiting the tumor progression, where individuals with high CXCL10 levels have a low risk for ovarian cancer progression (Bronger et al., 2016; Au et al., 2017). In line with this, our results observed that CXCL10 played a prior role in ovarian cancer immune characteristics and progress when compared to CXCL9. CXCL10 expression was regarded as a favorable prognostic biomarker and verified to have high predictive power for this risk classification. Meanwhile, the chemokine landscape of ovarian cancer was found to be quite heterogeneous, because of different functions of known lymphocyte-recruiting chemokines in TME, such as CCL2, CXCL9, CXCL10, CXCL12, and CXCL16 (Arnold et al., 2005; Zsiros et al., 2015; Lieber et al., 2018). Notably, CXCL10 is an important lymphocyte chemoattractant to mediate the cross-talk between cancer and immune cells (Zsiros et al., 2015; Mlynska et al., 2019). Consistent with the previous studies, CXCL10-positive tumors had higher antigen processing, antitumor immune response, and TIL accumulation (Au et al., 2017; Theodoraki et al., 2018; Unger et al., 2018), thereby suggesting that CXCL10 expression is a predictive biomarker to evaluate immune cell infiltration in ovarian cancer TME. According to our results, most high-risk individuals with low CXCL10 expression was negatively associated with immune cell infiltration degree. Moreover, compared with TME mismatch and stromal relevant signatures, integrated analysis revealed that TME immune-relevant signatures in four independent cohorts was significantly associated with CXCL10-related risk classification. Immune-relevant signatures (antigen-processing machinery, immune checkpoint, and T effector cells) presented in different risk classifications implicated that a higher CXCL10 expression was conducive to multiple TME immune regulation, especially the immune cell functions (Biragyn et al., 2004; Tokunaga et al., 2018). Furthermore, our data indicated that low-risk classification exhibited higher TME scores in all groups, which emphasized that CXCL10 activity is a core mechanism of TME immune regulation. Patients with advanced stage and poor OS were well characterized by the downregulation of the TME score. In line with our findings, some studies have indicated that the TME score possessed great value in predicting immunotherapeutic outcomes (Curtis et al., 2018; Ghisoni et al., 2019; Zeng et al., 2019).

In addition, through screening transcriptomic data, we depicted the prediction between CXCL10 and immune cell populations. We found a strong correlation between CXCL10 and antitumor immune cells, especially T cells, aDC, DCs, and Th2 cells (Eftimie et al., 2010; Drakes and Stiff, 2016; Singel et al., 2019; Yang et al., 2019). Moreover, there are multiple distinct immune TMEs that coexist within the patients and present heterogeneous fates in the clinical outcomes (Jimenez-Sanchez et al., 2017). The synergistic effect between CXCL10 and specific TME immune cells is one of the most important factors to prolong patient survival of ovarian cancer. Consistent with our finding, previous studies involved clinical specimens of ovarian cancer with TME antitumor immune cell recruitment and immunomodulatory molecule activity and revealed that immune cell accumulation was associated with good prognosis and therapy potential in ovarian cancer (Hatziveis et al., 2012; Coukos et al., 2016; Santoiemma et al., 2016; Au et al., 2017). Interestingly, CXCL10 not merely influences the immune cell infiltration but also is positively connected with antigen processing and T-cell metagene expression in TME (Au et al., 2017). Furthermore, we investigated the TME immune-cell signature gene statue between two CXCL10-related risk classifications. In our results, there are significantly distinct expression patterns and pathway enrichment in the two classifications. The high-risk group immune signature genes were mostly conducted through oncogenic KRAS signaling upregulation (Yang et al., 2018). However, the low-risk group was more likely to participate in IL2-STAT5 signaling, which showed the ability to initiate T-cell growth and differentiation (Kryworuchko et al., 2004). This observation may also help to facilitate the understanding of CXCL10 function in TME and development of precision immunotherapy.

Furthermore, the exact mechanisms of immune signature gene regulation are not well understood. Besides gene expression, some data indicated that tumor mutation burden (TMB) and SNP were closely associated with immune infiltration and immunotherapy effects (Bigley et al., 2018; Zhang et al., 2019; Jones et al., 2020). Through genomic alteration analysis, we provided insights into the somatically altered genes between two risk classifications. In our analysis, the low-risk group showed a different CNV compared to the high-risk group. As in a previous report, CNV is considered to be associated with various human cancers and caused by genomic rearrangement, such as deletion and duplication (Du et al., 2011; Zhang et al., 2016). In line with this theory, we observed that CXCL10-related immune signature gene alteration was obvious higher in the high-risk group. Consistent with previous studies on genomic instability in ovarian cancer (Vollebergh et al., 2012; Alkema et al., 2016; You et al., 2017; Tian et al., 2020), we observed that CXCL10-related immune signature genomic alteration was obviously higher in the high-risk classification. Another key finding from our study was that C > T transversions showed a higher spectrum in the high-risk classification compared with the low-risk one. Our result indicated that total alteration of ovarian cancers C > T transversion was associated with mutational signature 1, which is known as ultra-hypermutators involved in tumor (Essers et al., 2020). Previous reports (Spurdle et al., 2000; Goodman et al., 2005; Tan et al., 2018) and our data indicated that C > T transversion might increase genetic alteration, leading to ovarian cancer development owing to decreased TME immune cells. The identification of this mutational pattern may provide a new perspective to study the mechanism of chemokine-related TME formation and explanation of rising tumor incidence, as well as exploring individual mutations and their roles in cancer immunity and immunotherapy.

Our study has several limitations. Further verifying the prospective cohort of patients who received chemokine-based immunotherapy is necessary to conquer the deficiency of data. Moreover, the gene expression value is subjected to sampling bias due to the different platforms and individuals. In our study, not all patients with a high TME score and immune infiltration have better outcomes, and more TME factors should be incorporated into the methodology for improvement of accuracy.

In summary, our current study suggested the biological process and prognostic roles of CXCL10 in more than 1,000 cases of ovarian cancer. The difference of CXCL10 expression patterns could not be ignored as a factor that causes the heterogeneity and complexity of individual immune TME. The comprehensive evaluation of CXCL10 in TME may help to expand our understanding of chemokine-related TME immune characteristics and obtain more effective immunotherapy strategies in ovarian cancer.

## Materials and Methods

### Ovarian Cancer Datasets and Preprocessing

We retrospectively collected the ovarian cancer gene expression from the Gene-Expression Omnibus (GEO) and The Cancer Genome Atlas (TCGA) databases. In total, we gathered four cohorts with 1,673 samples from 10 public microarray datasets, including available follow-up time, survival status and international federation of gynecology and obstetrics (FIGO) stages. Patients without survival information were removed from further evaluation. For microarray data, we downloaded the raw data and clinical information from GEO repository^1^ by the GEOquery package (Davis and Meltzer, 2007), then normalizing (Affy package) expression values of all probes and combined the datasets with the same microarray platform. As for the datasets in TCGA, Affymetrix Human Genome U133A Array microarray data of gene expression was downloaded from the Genomic Data Commons (GDC,^2^) by using the R package TCGAbiolinks, which was specifically developed for integrative analysis with GDC data (Colaprico et al., 2016). The non-biological technical biases batch effects between different datasets within the same platform were adjusted by ComBat algorithm (Johnson et al., 2007). The somatic mutation data was acquired from TCGA database by using the maftools package (Mayakonda et al., 2018). The TCGA ovarian cancer dataset from Affymetrix SNP 6.0 at Genome Analysis Platform of the Broad Institute was downloaded for individual Copy Number Variation (CNV) analysis. Data was analyzed with the R (version 3.6.1) and R Bioconductor packages.

### TME Immune Signature Genes Definition

We used Bindea’s immune cells genes signature (Bindea et al., 2013), which is well known as a highly sensitive and specific discrimination of 23 human immune cells phenotypes, including mast cells, DCs, aDC, Natural killer cells (NKs), Macrophages, and T cells subsets, etc. Moreover, we constructed TME signature gene sets from the study of Mariathasan (Mariathasan et al., 2018), and it includes 14 TME categories according to different molecular and signaling pathway functions, such as immune-relevant signatures, mismatch-relevant signatures and stromal-relevant signatures.

### Estimation of Cell Infiltration in TME

To quantify the relative abundance of immune cell infiltration in ovarian cancer TME, we employed single-sample gene-set enrichment analysis (ssGSEA) algorithm that defined the enrichment score of immune cells genes set in each patient within a given dataset. In order to identify the immune cells were associated with CXCL10-determined risk classification, we grouped the cells into four distinct groups, such as Immune cluster-A, Immune cluster-B, Immune cluster-C, and Immune cluster-D. Immune cells differentially enriched scores were determined by using the limma R package (Ritchie et al., 2015), which implemented an empirical Bayesian approach to estimate score changes by using moderated *t*-tests.

### Generation of TME Signatures

To estimate the risk classification related ovarian cancer TME signature, we constructed a set of scoring system between two risk classifications of patients with ovarian cancer. The construction of TME signature was performed as follows: Two different risk classifications and all samples gene were extracted. Then, a consensus clustering algorithm was used to define TME-relevant genes signature, and principal component analysis (PCA) was conducted. Principal component 1 was extracted to serve as the genes signature score. After that, we evaluated the TME score by using a method similar to GGI (Sotiriou et al., 2006; Zeng et al., 2019).

TME score = ΣPC1_*i*_ − ΣPC1_*j*_

where i is the signature score of the low-risk classification, and j is the signature score of the high-risk classification.

### Pathway Enrichment Analysis for the Molecular Function

To further understand the genes function between two risk classification patterns, we performed a Gene set variation analysis (GSVA) with six immune cells gene sets. GSVA is a non-parametric and unsupervised method, which is generally used to evaluate the variation in pathway (KEGG) and biological process activity (GO) (Hanzelmann et al., 2013). Furthermore, we employed ClusterProfiler R package (Yu et al., 2012) to perform the risk classification associated pathways and biological processes, basing on adjusted expression data of all transcripts. We identified hallmark pathways among low to high risk and high to low infiltration individuals by using gene set enrichment analysis (GSEA) (Subramanian et al., 2005). The gene sets examined in pathway and biological process were obtained from MSigDB database of Broad Institute. Adjusted *P*-value < 0.05 was considered as statistically significance.

### Deciphering Alteration Pattern Operative in the Genome

The investigation of CNV was presented based on DNA profiling of individuals on Affymetrix SNP 6.0 Platform. We divided the segment file into two risk classifications and performed with the GISTIC 2.0 algorithm (Mermel et al., 2011). The somatic point, missense, insertion, and deletion mutations were annotated by using information from TCGA somatic mutation dataset. For the two classification mutation plots, we compared 45 immune-cell signature genes alteration levels of selected patients. Then, we employed the framework proposed by Kim et al. (2016) to extract mutational signature. This framework based on Bayesian variant non-negative matrix factorization and it can automatically determine the optimal number of mutational signatures. The mutation portrait matrix was factorized into non-negative matrices and corresponding mutational activities. The columns of non-negative matrix demonstrated the number of extracted alteration, and the rows indicated the 96 mutational contexts, which derived from a combination of six mutational types (i.e., C > A, C > G, C > T, T > A, T > C, and T > G) and their 5′ and 3′ adjacent bases (Dulak et al., 2013; Li et al., 2018). The corresponding mutational matrix revealed the individual mutation activities and the corresponding mutational signatures 1, 3, 6, 8, and 25. These mutational signatures were curated by the Catalog of Somatic Mutations in Cancer (COSMIC) Version 2 (COSMIC, ^3^).

### Statistical Analysis

In this study, statistical analysis was mainly performed by using R software and SPSS software (version 24.0). For the two groups comparison, statistical significance was evaluated by unpaired *t* tests. The Kruskal–Wallis tests were used to conduct difference comparisons of three or more groups. In the survival analysis, survival curves were generated *via* the Kaplan-Meier in each data set, and log-rank tests were utilized to determine significance of differences. The cut-off values for risk classification were evaluated based on the association between patient survival and CXCL10 expression in each cohort by using the survminer R package. The survminer can uninterruptedly screen all possible cut points to identify the maximum rank statistic. The associations between characteristics and OS were evaluated by Cox proportional hazard models. Correlation coefficients between TME infiltration score and expression of CXCL10 were computed by Spearman and distance correlation analyses. The waterfall plot of maftools package (Mayakonda et al., 2018) was applied to present the mutation landscape in patients with high and low risk ovarian cancer subtypes in standard cohort. All statistical *P* value were two-side, with *P* < 0.05 as statistically significance.

## Data Availability Statement

The data generated or analyzed during this study are available from the corresponding author upon reasonable request.

## Author Contributions

JJ and LZ conceived and drafted the manuscript. YL and TM drew the figures. QZ discussed the concepts of the manuscript, revised the manuscript, and approved the version to be submitted. All authors contributed to the article and approved the submitted version.

## Conflict of Interest

The authors declare that the research was conducted in the absence of any commercial or financial relationships that could be construed as a potential conflict of interest.

## Publisher’s Note

All claims expressed in this article are solely those of the authors and do not necessarily represent those of their affiliated organizations, or those of the publisher, the editors and the reviewers. Any product that may be evaluated in this article, or claim that may be made by its manufacturer, is not guaranteed or endorsed by the publisher.

## Footnotes

1
https://www.ncbi.nlm.nih.gov/geo/

2
https://portal.gdc.cancer.gov/

3
http://cancer.sanger.ac.uk/cosmic/signatures

## Supplementary Material

Supplementary Figure 1 Clustering of TME cells-infiltration in the Test cohorts. Unsupervised clustering of 23 TME immune types for ovarian cancer patients in the three independent test cohorts. CXCL10 expression, immune cluster, stages, grades and survival status are displayed as patient annotations. Hierarchical clustering was performed with Euclidean distance and Ward linkage.

Supplementary Figure 2 The mutational signatures selected immune signature genes. **(A)** The mutational activities of corresponding mutational signatures. **(B)** Alteration frequency ranked between high-risk classification (gray) and low risk classification (blue). The immune cells signature genes were showed under the X-axis. **c** Mutation frequencies were calculated based on average of each category (i.e., C > A, C > G, C > T, T > A, T > C, and T > G). We dichotomized the scores into low-and high-risk classification.

Supplementary Figure 3 TME signature characteristics with CXCL10-related risk classification and prognostic value. **(A)** Distribution of TME signatures in two different risk classifications of three independent test cohorts. **(B)** Kaplan–Meier curves of prognosis of TME scores in three independent test cohorts.

Supplementary Figure 4 TME scores related clinical parameters and immune cell infiltration in ovarian cancer. **(A)** Merged data of all four cohorts. And it showed that the distribution of TME scores were stratified by both CXCL10 risk classification (high/low) and TME infiltration cluster (high/low). The Kruskal-Wallis test was used to compare every two groups significantly. **(B)** Merged data of all four cohorts. Differences in TME scores among different pathological stages of ovarian cancer. The Kruskal-Wallis test was used to compare every two groups significantly. **(C)** Distribution of TME scores in different clinical outcomes of standard cohort, such as neoplasm grades, recurrence, invasion and copy number alteration (CNA).
