# Supplementary material for: Identification of CXCL10-Relevant Tumor Microenvironment Characterization and Clinical Outcome in Ovarian Cancer
Source: Front Genet. 2021 Jul 27;12:678747. doi: 10.3389/fgene.2021.678747 (PMC8354215; doi:10.3389/fgene.2021.678747)
Supplement: Supplementary Figure 1 — Clustering of TME cells-infiltration in the Test cohorts. Unsupervised clustering of 23 TME immune types for ovarian cancer patients in the three independent test cohorts. CXCL10 expression, immune cluster, stages, grades and survival status are displayed as patient annotations. Hierarchical clustering was performed with Euclidean distance and Ward linkage. [file Data_Sheet_1.PDF]

Figure S1

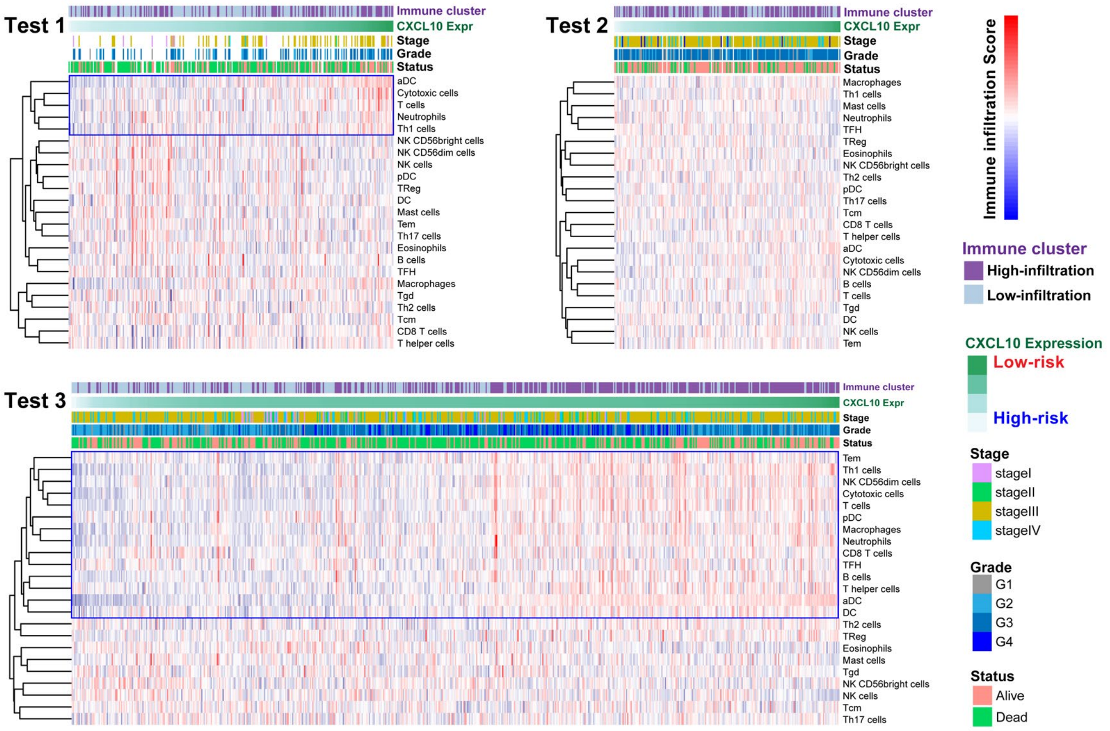

Figure S1. Clustering of TME cells-infiltration in the Test cohorts. Unsupervised clustering of 23 TME immune types for ovarian cancer patients in the three independent test cohorts. CXCL0 expression, immune cluster, stages, grades and survival status are displayed as patient annotations. Hierarchical clustering was performed with Euclidean distance and Ward linkage.

Figure S2

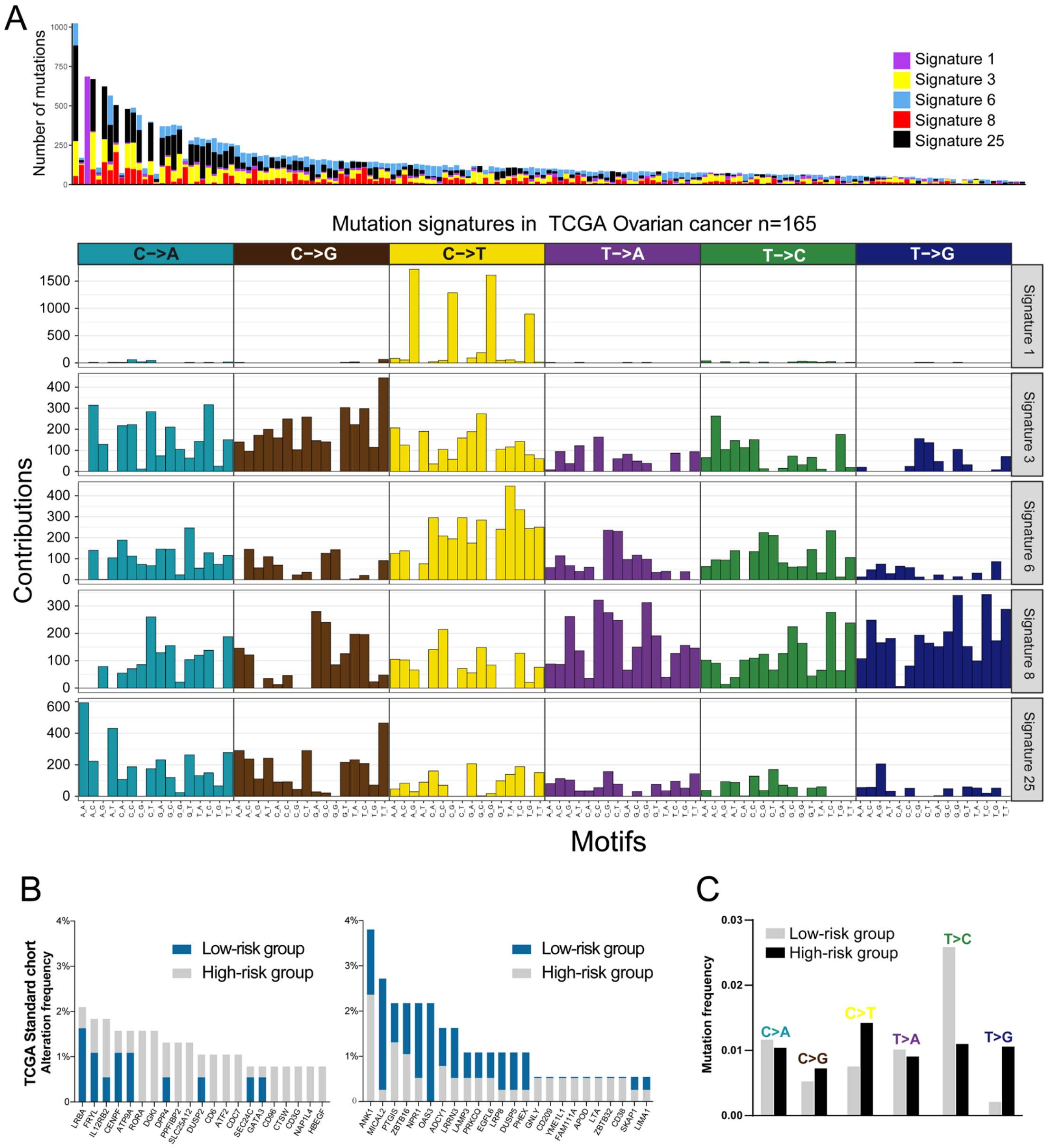

Figure S2. The mutational signatures selected immune signature genes. (A) The mutational activities of corresponding mutational signatures. **(B)** Alteration frequency ranked between high-risk classification (grey) and low risk classification (blue). The immune cells signature genes were showed under the X-axis. **c** Mutation frequencies were calculated based on average of each category (i.e., C>A, C>G, C>T, T>A, T>C, T>G,). We dichotomized the scores into low-and high-risk classification.

Figure S3

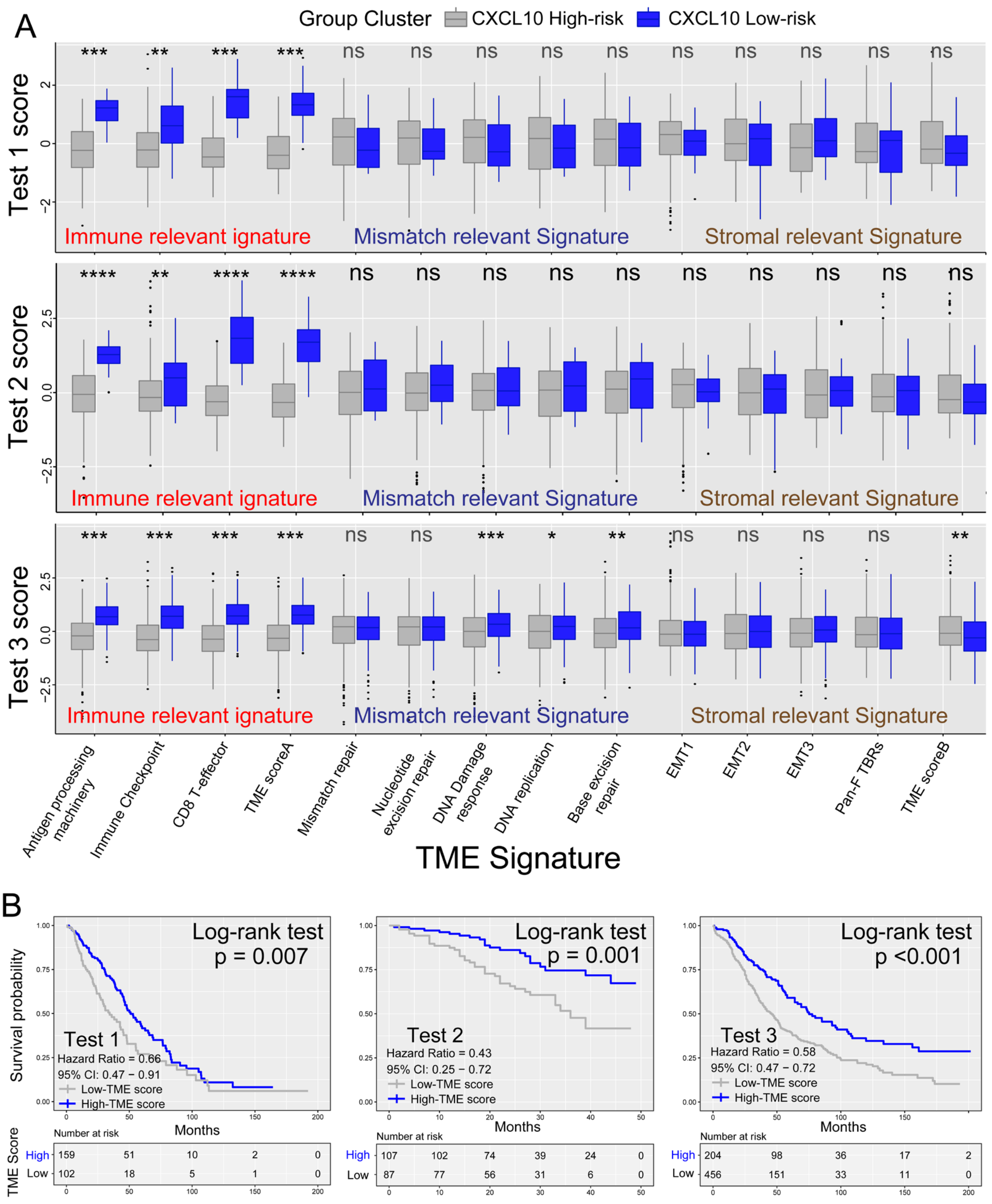

Figure S3. TME signature characteristics with CXCL10-related risk classification and prognostic value. (A) Distribution of TME signatures in two different risk classifications of three independent test cohorts. **(B)** Kaplan–Meier curves of prognosis of TME scores in three independent test cohorts.

Figure S4

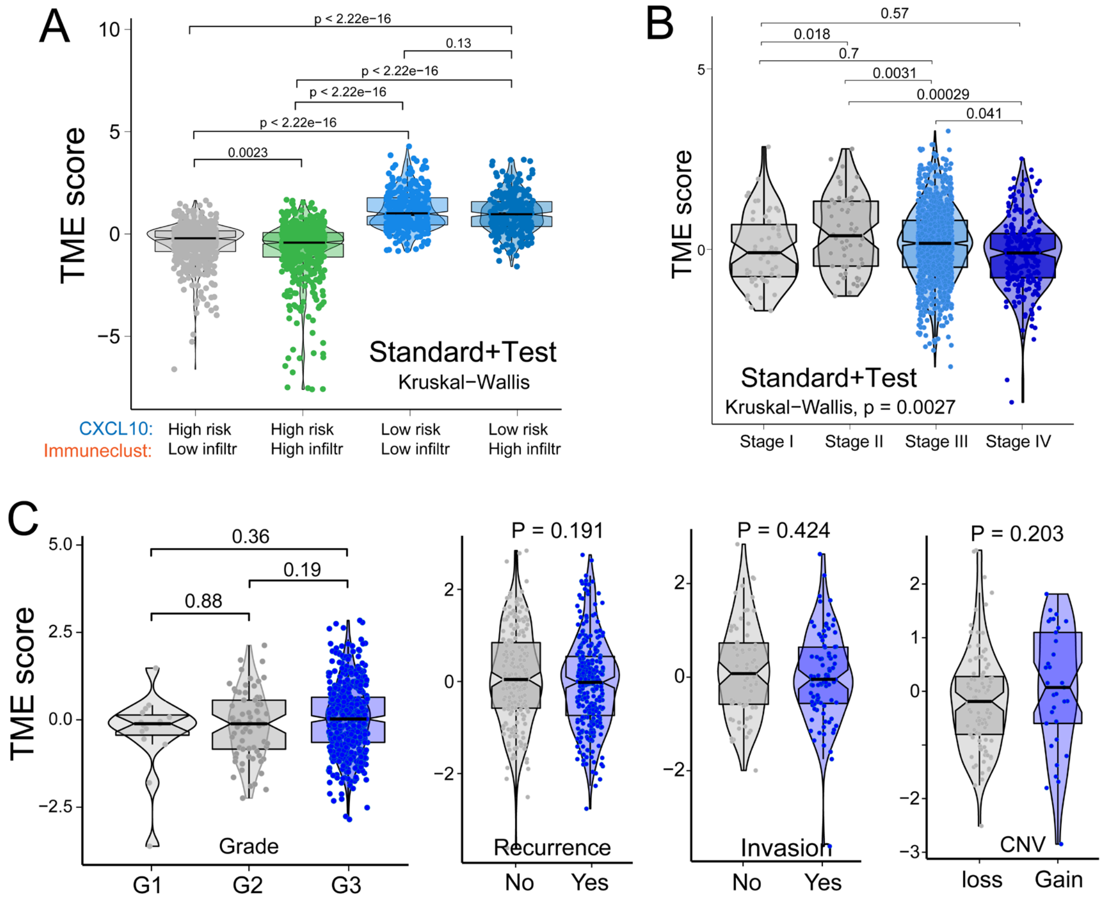

Figure S4. TME scores related clinical parameters and immune cells infiltration in ovarian cancer. **(A)** Merged data of all four cohorts. And it showed that the distribution of TME scores were stratified by both CXCL10 risk classification (high/low) and TME infiltration cluster (high/low). The Kruskal-Wallis test was used to compare every two groups significantly. **(B)** Merged data of all four cohorts. Differences in TME scores among different pathological stages of ovarian cancer. The Kruskal-Wallis test was used to compare every two groups significantly. **(C)** Distribution of TME scores in different clinical outcomes of standard cohort, such as neoplasm grades, recurrence, invasion and copy number alteration (CNA).
